# Inositol 1,4,5-trisphosphate-induced Ca^2+ ^signalling is involved in estradiol-induced breast cancer epithelial cell growth

**DOI:** 10.1186/1476-4598-9-156

**Published:** 2010-06-21

**Authors:** Cécilia Szatkowski, Jan B Parys, Halima Ouadid-Ahidouch, Fabrice Matifat

**Affiliations:** 1 Laboratoire de Physiologie Cellulaire et Moléculaire - JE-2530: Canaux ioniques et cancer du sein, Université d'Amiens, UFR des Sciences, 33 rue Saint-Leu 80039 Amiens, France; 2 Laboratory of Molecular and Cellular Signalling, Department of Molecular and Cellular Biology, Campus Gasthuisberg O/N1- bus 802 - K U Leuven, Herestraat 49, B-3000 Leuven, Belgium

## Abstract

**Background:**

Ca^2+ ^is a ubiquitous messenger that has been shown to be responsible for controlling numerous cellular processes including cell growth and cell death. Whereas the involvement of IP_3_-induced Ca^2+ ^signalling (IICS) in the physiological activity of numerous cell types is well documented, the role of IICS in cancer cells is still largely unknown. Our purpose was to characterize the role of IICS in the control of growth of the estrogen-dependent human breast cancer epithelial cell line MCF-7 and its potential regulation by 17β-estradiol (E_2_).

**Results:**

Our results show that the IP_3 _receptor (IP_3_R) inhibitors caffeine, 2-APB and xestospongin C (XeC) inhibited the growth of MCF-7 stimulated by 5% foetal calf serum or 10 nM E_2_. Furthermore, Ca^2+ ^imaging experiments showed that serum and E_2 _were able to trigger, in a Ca^2+^-free medium, an elevation of internal Ca^2+ ^in a 2-APB and XeC-sensitive manner. Moreover, the phospholipase C (PLC) inhibitor U-73122 was able to prevent intracellular Ca^2+ ^elevation in response to serum, whereas the inactive analogue U-73343 was ineffective. Western-blotting experiments revealed that the 3 types of IP_3_Rs are expressed in MCF-7 cells and that a 48 hours treatment with 10 nM E_2 _elevated IP_3_R3 protein expression level in an ICI-182,780 (a specific estrogen receptor antagonist)-dependent manner. Furthermore, IP_3_R3 silencing by the use of specific small interfering RNA was responsible for a drastic modification of the temporal feature of IICS, independently of a modification of the sensitivity of the Ca^2+ ^release process and acted to counteract the proliferative effect of 10 nM E_2_.

**Conclusions:**

Altogether, our results are in favour of a role of IICS in MCF-7 cell growth, and we hypothesize that the regulation of IP_3_R3 expression by E_2 _is involved in this effect.

## Background

Ca^2+ ^is a ubiquitous messenger that has been shown to be responsible for controlling numerous cellular processes including muscle contraction, exocytosis, gene expression, cell growth and cell death [1-3]. Numerous studies have shown that Ca^2+ ^is involved in the control of cellular growth through its interaction with a plethora of intracellular proteins and cellular transduction pathways. The Ca^2+^-dependent processes are often involved in highly important cellular responses that are strikingly exemplified by their role in life-and-death decisions. Consequently, Ca^2+ ^needs to be used in an appropriate manner to determine cell fate; if this balancing act is compromised, pathology may ensue [4]. In the case of malignant cells, the importance of Ca^2+ ^homeostasis has been demonstrated by studies showing that some highly phosphorylated inositol phosphates [5] and antagonists of the phosphoinositide pathway [6] or Ca^2+ ^influx [7] arrest the growth of a variety of tumour cells in culture [8]. Furthermore, it has been shown that Ca^2+ ^plays a central role in vitamin D-induced cell death in cancerous cells [9-11]. Free intracellular Ca^2+ ^is provided by two main sources: i) extracellular, through a variety of Ca^2+ ^entry channels, ii) intracellular, from the endoplasmic reticulum (ER), mainly through two types of intracellular Ca^2+ ^channels [i.e. inositol 1,4,5-trisphosphate (IP_3_) receptor (IP_3_R) and ryanodine receptor]. IP_3_R protein subtypes (namely IP_3_R1, IP_3_R2 and IP_3_R3) are encoded by three different genes in mammals but share high similarity in their primary sequences and are expressed to varying degrees in various cell types [12]. Even though they share common properties, it has been shown, however, that they are responsible for different types of Ca^2+ ^signals when expressed alone [13,14]. Temporal characteristics, that is amplitude, frequency and duration, of the Ca^2+ ^signal determine its intracellular function [15]. For example, it has been shown that the encoding of several genes is dependent on the oscillatory or transitory pattern of the Ca^2+ ^signal [16,17].

The steroid hormone 17β-estradiol (E_2_) is a key growth regulator involved in normal breast development where it stimulates growth of the ductal system. However, clinical and experimental data have clearly established that exposure to estrogens is the leading cause of sporadic female breast cancer [18]. The predominant biological effects of E_2 _have traditionally been considered to be based on its interaction with intracellular estrogen receptors [19]. These act via the regulation of transcriptional processes, involving nuclear translocation, binding to specific estrogen responsive elements and ultimately regulate gene expression [20-23]. Furthermore, E_2 _has also been shown to be involved in cellular responses that do not require the stimulation of estrogen receptors (i.e. alternative or non-genomic pathway) [24,25]. Whereas the involvement of IP_3_-induced Ca^2+ ^signalling (IICS) in the physiological activity of numerous cell types is well documented, the role of IICS in cancer cells is still largely unknown. In the case of breast cancer cells, only a few studies have described the Ca^2+ ^release mechanisms [26] and their potential modulation by E_2 _and anti-estrogens [27-30].

In this study, we investigate the potential involvement of E_2 _in regulating IICS in the estrogen-dependent MCF-7 cell line. We show that the expression level of IP_3_R3 is controlled by E_2 _in an estrogen receptor-dependent manner and that the growth of MCF-7 cells induced by E_2 _is sensitive to pharmacological inhibitors of IP_3_Rs. Furthermore, IP_3_R3 gene silencing using specific siRNA diminishes E_2_-induced cell growth and changed the temporal feature of ATP-induced intracellular Ca^2+ ^signals. We conclude that IICS is involved in E_2_-induced MCF-7 cell growth and that the regulation of IP_3_R3 expression could explain this effect.

## Results

### Serum and E_2 _trigger Ca^2+ ^release from IP_3_-sensitive stores

**Figure 1:**
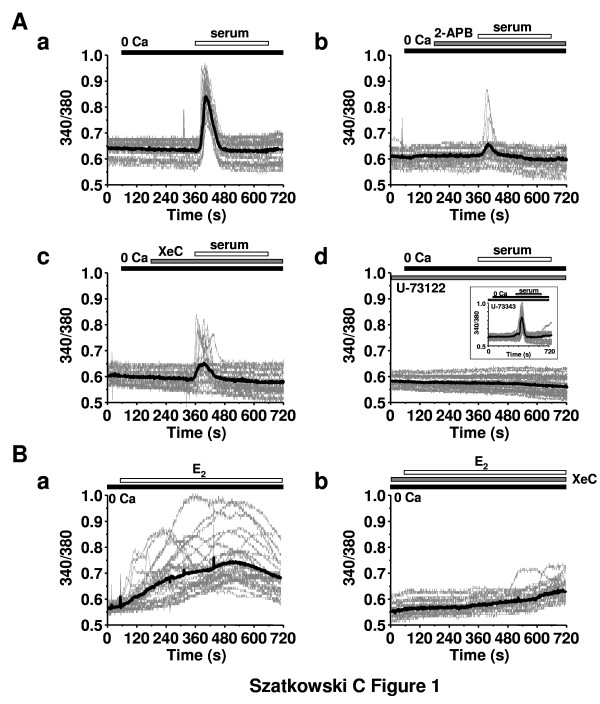
**Serum triggers an intracellular Ca^2+ ^signal**. (A) The perfusion of a Ca^2+^-free recording solution containing 5% of serum on Fura-2-loaded MCF-7 cells elicited a strong intracellular Ca^2+ ^signal (a). This signal was due to release from IP_3_-sensitive Ca^2+ ^stores as it was sensitive to previous application of 2-APB (75 μM, b) or XeC (25 μM, c). Furthermore, U-73122 (20 μM) prevented the effect of serum on intracellular Ca^2+ ^release (d) whereas the inactive analogue U-73343 (20 μM) was ineffective (d, inset). (B) E_2 _(10 nM) triggered intracellular Ca^2+ ^elevations in MCF-7 cells perfused with a Ca^2+^-free medium (a); these Ca^2+ ^elevations were inhibited by XeC (25 μM, b). In each panel, the results show the typical traces of 27 to 35 cells, always represented at the same scale; each time, also the mean signal is represented (thick black line).

As for many other cell types, the growth of MCF-7 cells is dependent on internal Ca^2+ ^and these cells are able to elicit intracellular Ca^2+ ^signals in response of multiple ligands [31]. When Fura-2-loaded MCF-7 cells were perfused in a Ca^2+^-free medium, serum was able to trigger Ca^2+ ^release (Figure 1Aa) from IP_3_-sensitive Ca^2+ ^pools since the internal Ca^2+ ^elevation was inhibited by 58.6 ± 15.3% (n = 56; P < 0.05) by the known IP_3_R antagonist 2-APB (75 μM; Figure 1Ab) [32]. In addition, the IP_3_R inhibitor XeC (25 μM) similarly inhibited serum-triggered IICS by 64.7 ± 11.7% (Figure 1Ac; n = 49; P < 0.05). To further assess the role of the IP_3_R in this process, we also used the phospholipase C inhibitor U-73122 (20 μM). The latter completely suppressed the serum-induced intracellular Ca^2+ ^release (Figure 1Ad) whereas the inactive analogue U-73343 (20 μM) was ineffective (Figure 1Ad, inset). Finally, we also tested whether E_2 _was able to induce intracellular Ca^2+ ^release from IP_3_-sensitive stores. Indeed, in the absence of extracellular Ca^2+^, E_2 _(10 nM) evoked Ca^2+ ^signals (Figure 1Ba) that were inhibited for 87.3 ± 10.2% (n = 52; P < 0.05) by 25 μM XeC (Figure 1Bb).

### Pharmacological inhibitors of IP_3_-induced Ca^2+ ^signalling inhibited MCF-7 cell growth

**Figure 2:**
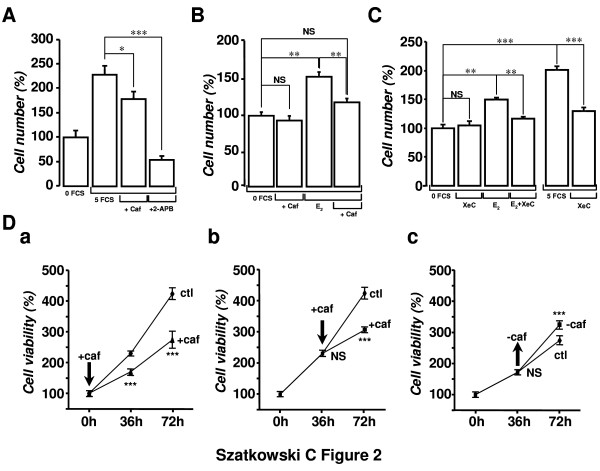
**Pharmacological inhibitors of IP_3_Rs inhibited 5-FCS- and E_2_-induced MCF-7 cell growth**. (A) The growth of MCF-7 cells induced by a 48 h treatment with 5-FCS was sensitive to pharmacological inhibitors of IP_3_R. Left bar graph shows the cell number obtained in 0-FCS and serves as a control experiment in order to estimate the proliferative effect of 5-FCS. Caffeine (500 μM) and 2-APB (75 μM) were both able to inhibit significantly 5-FCS-stimulated cell growth (P < 0.05 and P < 0.001, respectively). Values are the mean ± S.E.M. of 6 independent experiments. (B) Pharmacological inhibition of IP_3_R was responsible for the inhibition of E_2_-induced cell growth. Whereas caffeine (500 μM) was ineffective alone in control conditions, it inhibited the stimulation of cell growth by E_2 _(10 nM, P < 0.01). Values are the mean ± S.E.M. of 3 independent experiments. (C) XeC (10 μM) inhibited both 5-FCS and E_2_-induced MCF-7 cell growth. Values are the mean ± S.E.M. of 4 independent experiments. (D) Kinetics and reversibility of the inhibition by caffeine of E_2_-induced MCF-7 cell viability. Cells were starved for a 24 h period and were then stimulated by 10 nM E_2_. Caffeine (500 μM) was either added (+caf) at the beginning of the experiment (a), or after 36 h (b). In both cases, caffeine inhibited the E_2_-induced increase in cell viability (P < 0.001). The effect of caffeine was reversible since washout (-caf) of this compound after 36 h permitted to significantly restore the proliferative effect of E_2 _at 72 h (c). Arrows indicate the time of application (downward) or washout (upward) of caffeine. Values are the mean ± S.E.M. of 3 independent experiments.

In order to verify the possible involvement of IICS in MCF-7 cell growth, pharmacological inhibitors of IP_3_R (i.e. caffeine, 2-APB and XeC) were tested on 5-FCS- and E_2_-induced cell growth. As could be expected, cell counting using the trypan-blue exclusion method showed that 5-FCS was able to increase MCF-7 cell growth compared to a starvation culture medium (Figure 2A). Interestingly, both caffeine (500 μM) and 2-APB (75 μM) significantly inhibited cell growth in 5-FCS by 23.0 ± 6.2% (P < 0.05; n = 18) and 76.2 ± 5.4% (P < 0.001; n = 18), respectively. In all experiments, the total number of dead cells did not exceed 5%, thus the increase in cell number could be attributed to an increase of cell proliferation. Addition of E_2 _in the culture medium similarly stimulated cell growth (Figure 2B). We tested the effect of caffeine on E_2_-induced growth. In this latter case, MCF-7 cells were seeded in 0-FCS for a 24 h period in order to eliminate all proliferative agents and were then stimulated with E_2 _for 48 h in the absence or the presence of caffeine (500 μM). Figure 2B shows that caffeine was able to inhibit by 66.7 ± 3.1% the growth induced by 10 nM E_2 _(P < 0.01; n = 18). The cell growth in the presence of caffeine alone was not statistically different from that in 0-FCS (90.4 ± 5.5%, n = 18 vs 100.0 ± 6.8%, n = 18; P > 0.05). The effect of 2-APB could not be adequately tested as it triggered even under control conditions a significant elevation in cell death (>55%). Furthermore, as caffeine and 2-APB have been described as non-specific IP_3_R antagonists, we performed experiments using XeC, another IP_3_R inhibitor, in order to increase the weight of evidence in support of a role for IICS in stimulating cell proliferation. A concentration of 10 μM XeC was chosen in order to limit potential side effects of the compound during a long-term treatment; this concentration was verified to be sufficient to inhibit E_2_-induced Ca^2+ ^release (data not shown). Figure 2C shows that XeC (10 μM) inhibited by 69.3 ± 7.1% (P < 0.05, n = 9) and by 71.0 ± 6.5% (P < 0.001, n = 9) the proliferation induced by 10 nM E_2 _and 5-FCS, respectively.

Figure 2D demonstrates the kinetics and the reversibility of the inhibitory effect of caffeine on E_2_-induced cell growth. Addition of caffeine (500 μM) at the beginning of the experiment or at intermediate time (0 h and 36 h, Figure 2Da and 2b, respectively) reduced the increase of cell viability induced by E_2_. The inhibitory effect of caffeine on the E_2_-induced cell growth is 46.7 ± 8.3% (P < 0.001; n = 27) at 36 h and 47.9 ± 8.4% (P < 0.001; n = 27) at 72 h (Figure 2Da). In the same way, the inhibition is 36.5 ± 6.7% (P < 0.001; n = 27) at 72 h when caffeine was added at the intermediate time (Figure 2Db). On the contrary, wash-out of caffeine at the intermediate time point significantly restored the proliferative effect of E_2_; the cell growth was in this case indeed 31.3 ± 4.5% (P < 0.001; n = 27) higher compared to the condition in which caffeine was still present in the culture medium (Figure 2Dc).

### Expression of IP_3_Rs isoforms and their regulation by E_2_

**Figure 3:**
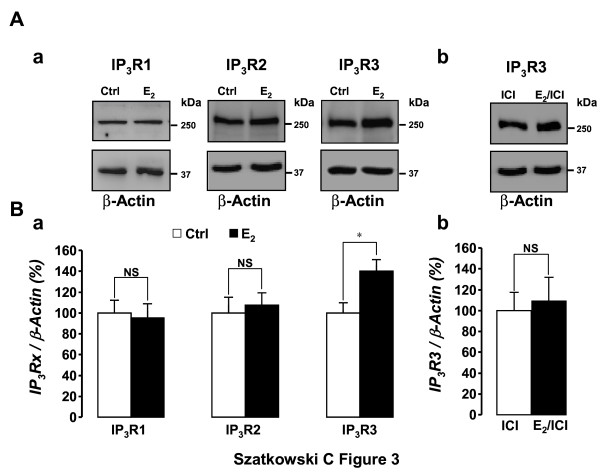
**Expression of IP_3_Rs isoforms and their regulation by E_2_**. (A) MCF-7 cells express the three IP_3_R isoforms. (a) Western blotting allowed the specific detection in MCF-7 microsomes (50 μg proteins/lane) of IP_3_R1, IP_3_R2 and IP_3_R3. Representative blots for 7 to 8 independent experiments are shown. Whereas E_2 _had no effect on the expression level of IP_3_R1 (left panel) and IP_3_R2 (middle panel), E_2 _increased the expression of IP_3_R3 (right panel, P < 0.05). This effect of E_2 _involves an estrogen receptor-dependent intracellular pathway since it was antagonized by the specific anti-estrogen ICI-182,780 (1 μM, b). (B) Statistical analysis of the effect of E_2 _on the expression level of the 3 types of IP_3_Rs (a) and of the inhibitory effect of ICI-182,780 on E_2_-induced stimulation of IP_3_R3 expression (b). In the presence or absence of ICI-182,780, the expression level of IP_3_R3 is not significantly different (113.4 ± 21.9 vs. 100.0 ± 18.2% in control conditions, n = 6).

Considering these results showing the implication of IICS in MCF-7 cell growth, we carried out western-blotting experiments on MCF-7 microsomes in order to characterize the types of IP_3_Rs expressed in these cells and the potential effect of E_2 _on their expression level. Figure 3 shows that the 3 types of IP_3_Rs are expressed. Whereas the expression level of IP_3_R1 and IP_3_R2 remained unchanged following a 48 h treatment with 10 nM E_2 _(Figure 3Aa, left and middle panel, respectively), E_2 _was able to elevate the expression level of IP_3_R3 (Figure 3Aa, right panel). Compared to control conditions, the expression level was 95.1 ± 14 (P > 0.05, n = 7) for IP_3_R1, 107.5 ± 12 (P > 0.05, n = 7) for IP_3_R2 and 140.2 ± 11.3% (P < 0.05; n = 8) for IP_3_R3 (Figure 3Ba). Furthermore, this latter effect was counteracted by the specific antagonist of the estrogen receptor, ICI-182,780 (1 μM; Figure 3Ab and 3Bb), a compound which is known to inhibit E_2_-induced MCF-7 proliferation [[33] and data not shown].

### Silencing of IP_3_R3 by RNA interference limits the proliferative effect of E_2_

**Figure 4:**
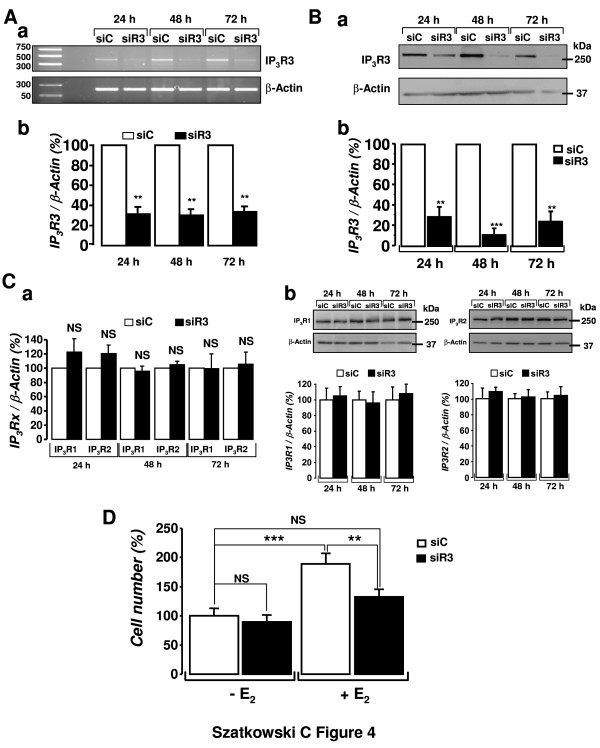
**Silencing of IP_3_R3 by RNA interference limits the proliferative effect of E_2_**. (A) Validation of the efficiency of the siRNAs used at the mRNA level (a, representative illustrations for 3 independent experiments). The IP_3_R3 mRNA level was lowered by about 70% (see text for details) at 24, 48 and 72 h post-transfection. (B) siR3 was responsible for a rapid and long-lasting diminution of the expression of IP_3_R3 at the protein level (a). Compared to control conditions, siR3 diminished the expression of IP_3_R3 at 24, 48 and 72 h (b). (C) Following IP_3_R3 silencing, the levels of IP_3_R1 and IP_3_R2 mRNA (a) and protein (b) were not significantly changed at 24, 48 and 72 h post-transfection. Representative blots for 3 to 4 independent western blotting experiments are shown. (D) MCF-7 cells were transfected with siC or siR3 and seeded for 18 h in 5-FCS and then starved for 6 h in 0-FCS. Cells were subsequently cultured in 0-FCS in the absence (-E_2_) of the presence (+E_2_) of E_2 _(10 nM) for 48 h. MCF-7 cell number was measured at 72 h post-transfection using the trypan-blue exclusion method. E_2_-induced increase in cell number was strongly diminished in siR3-transfected MCF-7 cells compared to siC-transfected cells (P < 0.01).

In order to further investigate the involvement of IP_3_R3 in E_2_-induced MCF-7 cell growth, the expression of IP_3_R3 in MCF-7 cells was silenced by the use of RNA interference. Figures 4A and 4B depict the efficiency of gene silencing at respectively the mRNA and the protein level following transfection of MCF-7 cells with a siRNA directed against IP_3_R3 (siR3) or a control siRNA (siC). At 24, 48 and 72 h post-transfection, siR3 reduced the IP_3_R3 mRNA by 68.7 ± 7.4% (n = 3, P < 0.01), 69.8 ± 6.3% (n = 3, P < 0.01) and 66.2 ± 5.7% (n = 3, P < 0.01), respectively. Also at the protein level, siR3 induced a similar decrease in IP_3_R3 expression (Figure 4Bb). The expression of the IP_3_R3 was diminished by 70.8 ± 9% (n = 3, P < 0.01), 88.6 ± 6.3% (n = 3, P < 0.001) and 75.4 ± 10.8% (n = 3, P < 0.01) at 24, 48 and 72 h respectively (Figure 4Bb). Treatment with siR3 had no effect on the expression levels of the other IP_3_R isoforms and no adaptation phenomenon occurred. Quantitative PCR experiments show that the level of IP_3_R1 and IP_3_R2 mRNA was not significantly changed (122.5 ± 18.3%; 95.9 ± 7.2%; 98.8 ± 21.1 for IP_3_R1 and 120.6 ± 11.9%; 105.0 ± 4.8%; 105.4 ± 16.7% for IP_3_R2 at 24, 48 and 72 h respectively, n = 3; Figure 4Ca). These latter results were confirmed by western-blotting experiments (Figure 4Cb). Compared to control, the level of IP_3_R1 and IP_3_R2 proteins was 105.0 ± 11.9%; 96.1 ± 14.2%; 108.2 ± 11.2 for IP_3_R1 and 108.9 ± 5.2%; 102.3 ± 9.8%; 104.4 ± 10.5% for IP_3_R2 at 24, 48 and 72 h, n = 4; respectively).

Subsequently, we tested the effect of siR3 on the E_2_-induced MCF-7 cell growth. Cells were transfected with either siC or siR3 and seeded in 5-FCS for 18 hours. After that, cells were starved for 6 h before being stimulated with 10 nM E_2 _for 48 hours. Whereas siR3 did not significantly modify the growth of MCF-7 cells in the absence of E_2 _(90.7 ± 9.1% of control; n = 27; P > 0.05), it appeared that siR3 was able to inhibit E_2_-induced increase in cell number by 63.2 ± 6.7% (n = 27, P < 0.01, Figure 4C). E_2 _stimulated MCF-7 cell growth by 89.4 ± 18% (n = 27, P < 0.001) in siC-transfected cells and only by 32.9 ± 12.6% (n = 27; P > 0.05) in siR3-transfected cells. Taken together, this strongly suggests that the Ca^2+ ^signal resulting from the activation of IP_3_R3 is at least partly involved in the proliferative effect of E_2_.

### IP_3_R3 silencing changed the temporal characteristics of intracellular Ca^2+ ^signalling

**Figure 5:**
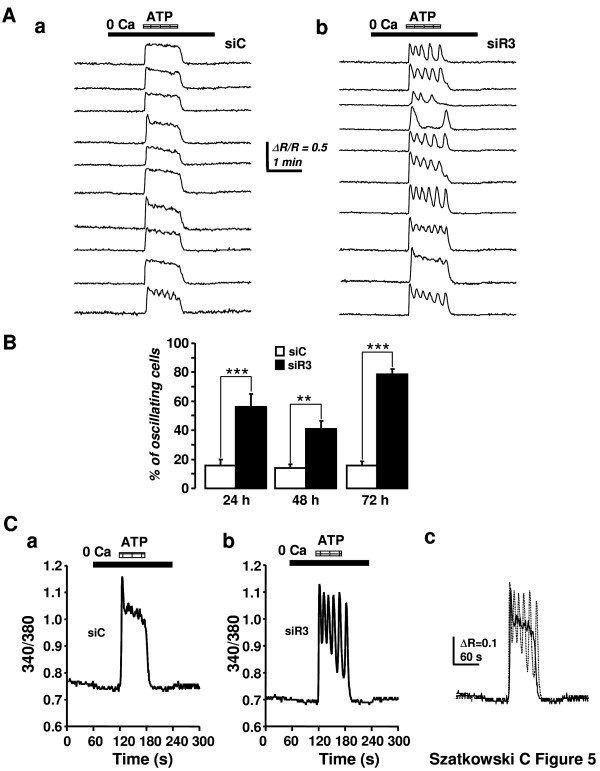
**IP_3_R3 silencing changed the characteristics of ATP-induced Ca^2+ ^signalling in MCF-7 cells**. (A) Typical ATP-induced Ca^2+ ^signals in MCF-7 cells 72 h after their transfection with siC (a) or siR3 (b). ATP (5 μM) was perfused in a Ca^2+^-free recording solution in order to avoid Ca^2+ ^entry. The Ca^2+ ^response changed from a plateau-type pattern to a characteristic oscillatory pattern in siR3-transfected cells. Representative for 6 independent experiments. (B) Statistical analysis of the effect of IP_3_R3 silencing at 24, 48 and 72 h post-transfection on the percentage of oscillating cells in response to 5 μM ATP in a Ca^2+^-free recording solution. IP_3_R3 gene silencing resulted, at any time, in a strong augmentation of the percentage of cells that respond to ATP by an oscillatory Ca^2+ ^signal. (C) Typical ATP-induced Ca^2+ ^signals recorded in a Ca^2+^-free medium 72 h after transfection with siC (a) or siR3 (b). Ca^2+ ^signals presented in a (full line) and b (dotted line) were superimposed in order to clearly show the modification of the pattern of the Ca^2+ ^response (c).

We then determined the effect of IP_3_R3 silencing on the intracellular Ca^2+ ^signals in response to ATP. ATP delivers very reproducible, standardized Ca^2+ ^signals that could be easily analyzed and quantified; furthermore, ATP is also able to stimulate MCF-7 proliferation [34]. Typical Fura-2 traces obtained after the perfusion of the cells with ATP (5 μM) in a Ca^2+^-free medium after 72 hours of transfection with either siC or siR3 are depicted in Figure 5A. Decreased IP_3_R3 levels provoked a drastic change in the characteristics of the ATP-induced Ca^2+ ^signal. Indeed, Ca^2+ ^signals changed from a plateau-type of response to a sinusoidal oscillatory-shaped signal. Statistical analysis revealed that at 24, 48 and 72 h post-transfection, the number of oscillating cells in response to ATP was much higher in siR3-transfected cells compared to siC-transfected cells (Figure 5B). The respective percentages of oscillating cells at 24, 48 and 72 h in siC-transfected cells versus siR3-transfected cells are 15.6 ± 4.4% (n = 6) vs 56.1 ± 8.9% (n = 6, P < 0.001); 13.9 ± 2.5% (n = 6) vs 41.1 ± 5.4% (n = 6, P < 0.01) and 15.1 ± 2.5% (n = 6) vs 78.3 ± 3.9% (n = 6, P < 0.001). In order to measure and compare the elevation of internal Ca^2+ ^concentration in siC- and siR3-transfected MCF-7 cells, we calculated the "area under curve" (AUC) for each trace. Figure 5C represents typical Ca^2+ ^signals measured at 72 h post-transfection in both conditions after perfusion with 5 μM ATP in a Ca^2+^-free medium. Superimposition of both traces clearly suggests that despite the pattern of the Ca^2+ ^signal was changed, the global amount of Ca^2+ ^released into the cell remained the same. This latter point was confirmed following statistical analysis. Indeed, the mean AUC values for Ca^2+ ^signals elicited in siC-transfected cells versus siR3-transfected cells were not statistically different (Table 1) whatever the time post-transfection tested (24, 48 and 72 h).

**Table 1:** IP_3_R3 silencing does not modify the AUC of intracellular Ca^2+ ^signals

Time post-transfection	siRNA	Mean AUC ± SD (n)	P value
**24 h**	**siC**	16.8 ± 6.5 (77)	0.392
		
	**siR3**	17.1 ± 6.5 (88)	

**48 h**	**siC**	18.7 ± 4.5 (79)	0.112
		
	**siR3**	19.5 ± 3.9 (83)	

**72 h**	**siC**	16.6 ± 5.9 (89)	0.125
		
	**siR3**	17.5 ± 4.2 (90)	

Statistical analysis of the effect of IP_3_R3 gene silencing on the AUC of Ca^2+ ^signals elicited by ATP (5 μM) in Ca^2+^-free medium. At any time after the transfection, the global amount of Ca^2+ ^released as assessed from the AUC appears unchanged between the cells treated with siC or siR3.

### IP_3_R3 silencing does not modify the sensitivity of IICS

**Figure 6:**
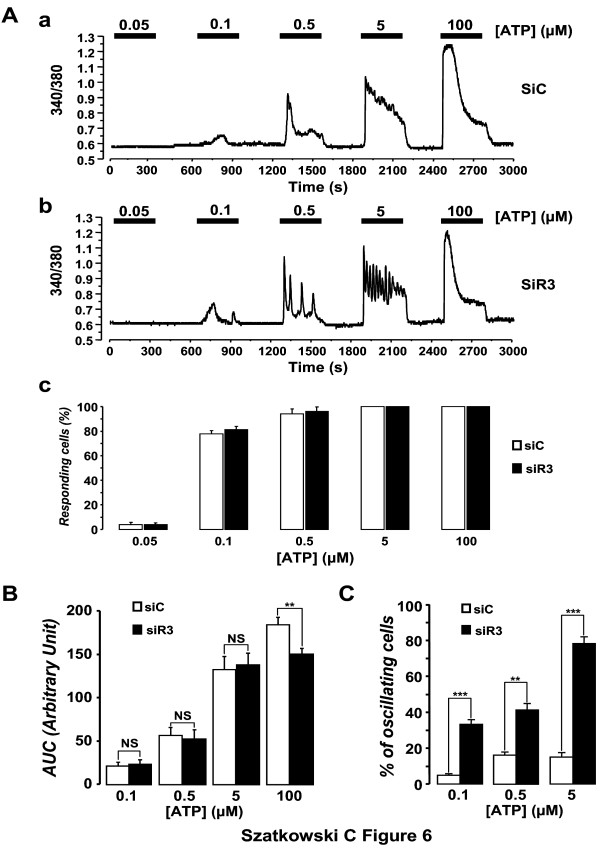
**IP_3_R3 silencing does not modify the sensitivity of the Ca^2+ ^release process**. (A) Typical Ca^2+ ^signals induced by ATP (50 nM - 100 μM) in Fura-2-loaded MCF-7 cells 72 h after their transfection with siC (a) or siR3 (b). ATP triggered intracellular Ca^2+ ^signals with a concentration threshold of about 100 nM in both cell batches but IP_3_R3 silencing was responsible for a change in the shape of the signal. The percentage of responding cells in siC- and siR3-transfected cells is unchanged whatever the concentration of ATP used (c). Results are representative of 8 independent experiments. (B) The magnitude of the signal is compared at various ATP concentrations (100 nM - 100 μM) and at the maximal concentration of ATP used, the magnitude of the signal is significantly decreased by about 20% (P < 0.01). (C) The number of oscillating cells was investigated at ATP concentrations ranging from 100 nM to 5 μM, and at each concentration the percentage of oscillating cells was largely greater in siR3-transfected cells (P < 0.01 or 0.001).

To further uncover how calcium signalling is affected by down regulation of IP_3_R3 expression, the sensitivity of the calcium release process and the magnitude of the calcium release at maximal agonist concentration were investigated (Figure 6). We have therefore performed experiments using different ATP concentrations (ranging from 50 nM to 100 μM) in control (siC-transfected, Figure 6Aa) and in siR3-transfected MCF-7 cells (Figure 6Ab), 72 h after the transfection. Figure 6A clearly demonstrates that the sensitivity of the Ca^2+ ^release process remains virtually unchanged since the threshold for ATP (about 100 nM) is the same for both types of cells. Furthermore, the percentage of responding cells is unchanged in siC vs siR3-transfected cells (Figure 6Ac). Values are 4.1 ± 0.9% vs 3.8 ± 0.8% (P > 0.05); 78.2 ± 3.1% vs 81.9 ± 2.8% (P > 0.05) and 94.3 ± 4.2% vs 95.5 ± 3.8% (P > 0.05) at 0.05, 0.1 and 0.5 μM ATP, respectively. For higher ATP concentrations (i.e. 5 and 100 μM) all the cells were responsive. However, the magnitude of the Ca^2+ ^signal (measurement of the AUC) is significantly diminished by 18.4 ± 5.7% (n = 76, P < 0.01; Figure 6B) at maximal ATP concentration. Indeed, the mean AUC (arbitrary unit) is 21.4 ± 4.4 vs 22.3 ± 4.9 at 100 nM ATP; 54.2 ± 11.7 vs 49.6 ± 12.2 at 500 nM ATP; 132.8 ± 15.2 vs 138.1 ± 13.5 at 5 μM ATP; and 184.4 ± 8.5 vs 150.5 ± 6.5 at 100 μM ATP, for siC- versus siR3-transfected cells, respectively. Finally, our results clearly demonstrate that the main difference between the cell types is the dramatic increase in the percentage of cells demonstrating an oscillating Ca^2+ ^signal pattern at all submaximal ATP concentrations after IP_3_R3 down regulation (Figure 6C): the mean percentage of cells demonstrating an oscillating pattern increased from 5.1 ± 0.9% to 33.3 ± 2.7% at 100 nM ATP; from 16.1 ± 1.9% to 41.4 ± 3.5% at 500 nM ATP and from 15.2 ± 2.5% to 78.3 ± 3.9% at 5 μM ATP, at each concentration in siC and siR3-transfected cells, respectively.

## Discussion

We showed in this study that MCF-7 cells express the 3 IP_3_R isoforms and that intracellular Ca^2+ ^release through these channels plays a role in the control of the growth of these cells. Indeed, using both pharmacological inhibitors and specific small inhibitory RNAs, we showed that IICS is involved in the increase in cell growth in response to addition of serum or E_2_. Our results are in agreement with previous studies showing that intracellular Ca^2+ ^elevation following ER emptying is crucial in order to ensure the activation by E_2 _of various protein kinases involved in cell cycle, such as mitogen-activated protein kinase, and to trigger MCF-7 cell proliferation [35]. In the same way, numerous studies have shown that IICS was responsible for stimulating the proliferation of various cell types [2] such as cerebral artery smooth muscle cells [36] and mouse cholangiocytes [37]. It has also been shown in gastric cancer cells that 2-APB inhibits cell proliferation and that IP_3_R3 belongs to genes that are over expressed in the case of peritoneal dissemination [38]. In the case of breast cancer, a few studies have shown that IP_3_R could be the target of a variety of proteins such as Bcl-2 and cyclins that might affect cell viability by respectively suppressing apoptosis [29,30] and probably stimulating proliferation [39]. Interestingly, this study demonstrates that IP_3_R3 expression is up regulated by E_2_. Moreover, this regulation occurs in an estrogen receptor-dependent manner since it was sensitive to ICI-182,780, a compound known to inhibit E_2_-induced MCF-7 cell proliferation [33]. As the proliferative effect of E_2 _involves IICS, we hypothesized that this could be, at least in part, due to the increased IP_3_R3 expression. This result is strengthened by the numerous studies showing a potential regulation of the expression level of IP_3_Rs by many factors, such as retinoic acid, TGF-β or phorbol esters [see 40 for review]. In particular, expression of IP_3_R isoforms has been shown to be controlled by steroids such as progesterone and E_2 _[41-43] or glucocorticoids [44]. Furthermore, an increased expression of IP_3_Rs has been described in proliferating arterial smooth muscle cell [45]. Other studies have already shown that the relative expression of the different IP_3_R isoforms is responsible for generating various Ca^2+ ^signals in term of duration, amplitude and shape (i.e. transient or oscillatory) [13]. For example, it has been shown that IP_3_R3 functions as an anti Ca^2+^-oscillatory unit in DT-40 cells [13] and in HeLa and COS-7 cells [14]. In full agreement with this, we demonstrated, on the basis of the results obtained using siRNA, that changing the IP_3_R3 levels in MCF-7 cells drastically changed the characteristics of the Ca^2+ ^signals. Importantly, no changes occurred in the sensitivity of the Ca^2+ ^signals to ATP after down-regulation of IP_3_R3, which is probably due to the expression of IP_3_R1 and IP_3_R2 which have a higher affinity for IP_3 _[13]. At maximal ATP stimulation, a small decrease in total Ca^2+ ^release was observed, but the largest difference was the profound increase (3- to 7-fold, depending on the ATP concentration used) in the number of cells displaying a pattern of sinusoidal Ca^2+ ^oscillations instead of a plateau phase. This fully supports the hypothesis that E_2 _partly controls MCF-7 cell growth by encoding specific Ca^2+ ^signals through the IP_3_R3. A relation between Ca^2+ ^oscillation frequency and transcription factors has already been shown [16,17]. It can therefore be hypothesized that the decrease in cell proliferation following IP_3_R3 silencing could be related to the modification of the temporal feature of the Ca^2+ ^signal.

Our results obtained following IP_3_R3 silencing show that basal MCF-7 proliferation in serum-deprived medium is not affected. This is due to the fact that in those conditions there is no factor present that can trigger internal Ca^2+ ^release. We have previously observed [24] a similar phenomenon in MCF-7 cells where iberiotoxin, an inhibitor of the voltage- and Ca^2+^-dependent K^+ ^channel BK, could impair the proliferation induced by E_2 _but not in basal conditions (0 FCS). Indeed, in the latter condition, basal Ca^2+ ^activity is probably too low to ensure the activation of the BK channels while after induction with E_2_, the internal Ca^2+ ^level is sufficiently elevated to activate these channels and therefore to uncover the sensitivity to iberiotoxin.

Interestingly, our study demonstrates a link between IP_3_R3 expression and cellular proliferation, though IP_3_R3 has also been previously implicated in cell death [46]. It is thought that IP_3_Rs and IICS can convey and/or enhance cell death signals by allowing for an efficient Ca^2+ ^shuttling between ER and mitochondria leading to mitochondrial Ca^2+ ^overload (see [47] for review). This efficient shuttling is only possible when the IP_3_R is closely apositioned to the mitochondria through physical interaction e.g. with the mitochondrial voltage-dependent anion channel. Several proteins can participate in this interaction, including glucose-regulated protein 75 and the sigma receptor Sig-1R [47]. This increased apoptosis does not seem to occur in the MCF-7 cells, what may be due either to the nearly complete absence of Sig-1R in those cells [48], or to additional regulation limiting the extent of Ca^2+ ^release and Ca^2+ ^transfer into mitochondrion by anti-apoptotic proteins as protein kinase B [49] or Bcl-2 [50]. Interestingly, the expression of the latter protein is up regulated by E_2 _in MCF-7 cells [51].

These various mechanisms may explain why, even though IP_3_R3 expression is increased in response to E_2_, apoptosis is not stimulated. The increased proliferation can therefore be due to each or both of the following elements, (1) a stimulation of cell metabolism and ATP production by a low to intermediate flux of Ca^2+ ^to the mitochondria, large enough to stimulate the Ca^2+^-sensitive mitochondrial dehydrogenases but not to cause detrimental effects and (2) Ca^2+ ^signals with a temporal pattern able to activate more efficiently transcription factors acting on the expression of genes involved in proliferation.

In conclusion, our observations indicate that the growth of MCF-7 human breast cancer cells induced by E_2 _is sensitive to pharmacological inhibitors of IP_3_Rs. Moreover, E_2 _treatment induced an upregulation of IP_3_R3 in an estrogen receptor-dependent manner while IP_3_R3 gene silencing affected both intracellular Ca^2+ ^signalling and cellular proliferation. Taken together, these results are suggestive in MCF-7 cells for a regulation of cell growth by specific Ca^2+ ^signals, but further work is needed to elucidate the precise mechanism(s) involved.

## Materials and Methods

### Cell culture

The MCF-7 cell line was purchased from the American Type Culture Collection (ATCC^® ^HTB-22™, LGC Promochem) and cells were used for a maximum of 10 passages after receipt or resuscitation. Cells were grown in an atmosphere saturated with humidity at 37°C and 5% CO_2 _in Eagle's Minimal Essential Medium supplemented with 2 mM L-glutamine, 0.06% HEPES Buffer and a mixture of penicillin (50 UI/ml)/streptomycin (50 μg/ml). In addition, the culture medium was either supplemented with 5% FCS (5-FCS) or not supplemented with FCS (0-FCS) and was renewed every two days.

### Cell viability

For cell growth assays, 75,000 MCF-7 cells were seeded in Petri dishes (diameter 60 mm) in 5-FCS. After 48 h, cells were incubated in a phenol-red-free 0-FCS for a 24 h starvation period. Cells were then washed and incubated with E_2 _(10 nM) or 5-FCS, alone or in association with caffeine (500 μM) or 2-APB (75 μM). After 2 days of treatment, the cell number was determined by trypan blue exclusion method. The counts were replicated six times and the experiments were repeated at least three times. Alternatively, cell viability was measured by the use of the 3-(4,5-dimethylthiazole-2-yl)-2,5-diphenyltetrazolium bromide reduction assay (MTT-assay) [52]. In brief, MCF-7 cells were plated in 6-well plates at 5.10^4 ^cells per well and allowed to grow for 48 h. Cells underwent a 24 h starvation in 0-FCS and were then stimulated with 10 nM E_2 _in the presence or the absence of caffeine (500 μM) for 36 h and 72 h. On the day of assay, treatment medium was replaced with medium containing 0.5 mg/ml of MTT and incubated for 1 h at 37°C. Medium was then aspirated off and 800 μl of DMSO was added to solubilise crystals. The optical density of each sample was read on a microplate reader (MRX II; Revelation software 4.22) at 570 nm against a blank prepared from cell-free wells.

### Ca^2+ ^imaging

MCF-7 cells were cultured at 5.10^4 ^cells per dish on glass cover slips and cells were loaded for 1 h with Fura-2/AM (3 μM in saline solution) at 37°C in a CO_2 _incubator and subsequently washed three times with the dye-free recording solution. The cover slip was then transferred into a perfusion chamber of a Zeiss inverted microscope equipped for fluorescence. Fluorescence was excited at 340 and 380 nm alternately, using a monochromator (Polychrome IV; TILL Photonics), and captured by a Cool SNAP HQ camera (Princeton Instruments) after filtration through a long-pass filter (510 nm). Background fluorescence was determined at 340 and 380 nm from an area of the cover slip free of cells. These values were routinely subtracted. Metafluor software (v.6.2; Universal Imaging, West Chester, PA) was used for acquisition and analysis. All recordings were carried out at room temperature (RT; 20-22°C). The cells were continuously perfused with the saline solution, and chemicals were added via the perfusion system. The flow rate of the whole-chamber perfusion system was set at 10 ml/min, and the chamber volume was 1 ml. Recording solution had the following composition (in mM): NaCl 145, KCl 5, CaCl_2 _2, MgCl_2 _1, and Hepes 10 at pH 7.4 (NaOH). In experiments where Ca^2+^-free solution was used, Ca^2+ ^was omitted and EGTA (1 mM) was added to the solution. The "Area Under Curve" (AUC) was calculated using OriginPro v.8 and permitted to measure the global amount of Ca^2+ ^released into the cells following stimulation.

### Western Blotting

Cells were washed twice with phosphate-buffered saline (PBS) and lysed by addition of RIPA buffer (200 μl/60 mm dish) containing protease inhibitor cocktail (Sigma P8340, 8 μl/ml). After 30-45 min incubation on ice, the cell lysates were scraped off the Petri dish and transferred to 1.5 ml tubes. The extracts were then centrifuged at 10,000 × g for 10 min at 4°C in a table-top centrifuge and the supernatants were saved for analysis. For the determination of the effect of E_2 _on the expression level of the various IP_3_R isoforms, microsomal preparations from MCF-7 cells were performed according to an earlier published procedure [53]. Protein concentration was determined using the BCA method and the amount of lysates or of microsomes corresponding to 50 μg of protein was denatured with SDS sample buffer and separated on 4-15% precast SDS-polyacrylamide gels (Bio-Rad). Proteins were then transferred overnight at 4°C to Immobilon-P PVDF membranes (0.6 mA/cm² constant current; Bio-Rad) in Tris-glycine buffer without methanol. Transfer membranes were incubated for 1 h at RT in Tris-buffered saline containing 0.1% Tween 20 (TBS-T) and 5% dry milk and then incubated overnight at 4°C in primary antibodies: goat anti-β-actin (1/2,000; Santa Cruz) as loading control, rabbit anti-IP_3_R1 (Rbt03, 1/1,000) [54]; rabbit anti-IP_3_R2 (CT2, 1/30) [55]; purified mouse anti-IP_3_R3 (610313; 1/2,000; BD Bioscience). Following primary antibody probing, the membranes were washed three times with TBS-T and incubated for 1 h at RT with the respective secondary horseradish-peroxidase-conjugated antibodies (Santa Cruz): anti-mouse (1/5,000) was used for the detection of IP_3_R3, anti-rabbit (1/5,000) for IP_3_R1 and IP_3_R2 and anti-goat (1/5,000) was used for the detection of β-actin. Proteins were visualized using the enhanced chemiluminescence system (Amersham) on a Chemidoc Apparatus and quantification was realized using Quantity One software.

### Total RNA isolation, reverse transcription of RNA and PCR experiments

Total RNA from MCF-7 cells was extracted by the Trizol-phenol-chloroform (Sigma Aldrich) procedure, including DNAse I treatment (0.2 U/μl, 30 min at 37°C, Promega). Total RNA was then reverse-transcribed into cDNA using oligodT primers and MultiScribe™ reverse transcriptase (Applied Biosystems). PCR experiments were carried out on an iCycler thermal cycler (Bio-rad) using Taq DNA polymerase (Invitrogen). PCR products were analysed by electrophoresis on 1.5% agarose gel and visualized by ethidium bromide staining. Finally, PCR products were quantified using Quantity One software and expressed as the ratio of IP_3_Rs on β-actin reference gene.

### Cell transfection

MCF-7 cells were collected after trypinization and submitted to electroporation using a Gene Pulser^® ^apparatus according to the manufacturer's instructions. Briefly, 2.10^6 ^cells were transfected with 2 μg siRNA directed against the human IP_3_R3 mRNA sequence (ON-TARGETplus, Dharmacon) or control siRNA (siGENOME non-targeting siRNA; Dharmacon). After the electroporation (program E-14), 500 μl of prewarmed culture medium were added and cells were transferred to a 1.5 ml tube and placed at 37°C for 15 min in a CO_2 _incubator. After that, cells were seeded in Petri dishes (diameter 60 mm). 18 h later, cells were treated for 6 h in 0-FCS and were then stimulated with E_2 _(10 nM) for 48 h.

### Statistical analysis

Results were expressed as mean ± S.E.M. Experiments were repeated at least three times. The Student's t-test was used to compare treatment means with control means. Statistical significance is indicated in the figures (NS, not significant; * P < 0.05; ** P < 0.01; *** P < 0.001).

### Reagents

All the products were from Sigma (France) unless otherwise stated. Final concentrations were obtained by appropriate dilution of stock solutions so that the solvent never exceeded 1/1,000.

## Abbreviations used

IP_3_: inositol 1,4,5-trisphosphate; IP_3_R: IP_3 _receptor; IICS: IP_3_-induced Ca^2+ ^signalling; E_2_: 17β-estradiol; 2-APB: 2-aminoethoxydiphenyl borate; XeC: xestospongin C; FCS: foetal calf serum; 5-FCS: culture medium containing 5% FCS; 0-FCS: serum-deprived culture medium; ER: endoplasmic reticulum.

## Competing interests

The authors declare that they have no competing interests.

## Authors' contributions

CS performed experiments and analysed the data. JBP and HOA participated in the design of the study and helped to draft the manuscript. FM conceived and performed experiments, analysed the data and drafted the article. All authors read and approved the paper.
